# Designing Online Interventions in Consideration of Young People’s Concepts of Well-Being: Exploratory Qualitative Study

**DOI:** 10.2196/10106

**Published:** 2019-01-30

**Authors:** Megan Winsall, Simone Orlowski, Gillian Vogl, Victoria Blake, Mariesa Nicholas, Gaston Antezana, Geoffrey Schrader, Niranjan Bidargaddi

**Affiliations:** 1 Personal Health Informatics Flinders University Clovelly Park Australia; 2 Partners Health Care Boston, MA United States; 3 Young and Well Cooperative Research Centre Abbotsford, Victoria Australia; 4 ReachOut Australia Sydney Australia; 5 Murdoch University Perth Australia; 6 Country Health South Australia Adelaide Australia

**Keywords:** well-being, youth, online intervention, participatory design, technology

## Abstract

**Background:**

A key challenge in developing online well-being interventions for young people is to ensure that they are based on theory and reflect adolescent concepts of well-being.

**Objective:**

This exploratory qualitative study aimed to understand young people’s concepts of well-being in Australia.

**Methods:**

Data were collected via workshops at five sites across rural and metropolitan sites with 37 young people from 15 to 21 years of age, inclusive. Inductive, data-driven coding was then used to analyze transcripts and artifacts (ie, written or image data).

**Results:**

Young adults’ conceptions of well-being were diverse, personally contextualized, and shaped by ongoing individual experiences related to physical and mental health, along with ecological accounts acknowledging the role of family, community, and social factors. Key emerging themes were (1) positive emotions and enjoyable activities, (2) physical wellness, (3) relationships and social connectedness, (4) autonomy and control, (5) goals and purpose, (6) being engaged and challenged, and (7) self-esteem and confidence. Participants had no difficulty describing actions that led to positive well-being; however, they only considered their own well-being at times of stress.

**Conclusions:**

In this study, young people appeared to think mostly about their well-being at times of stress. The challenge for online interventions is to encourage young people to monitor well-being prior to it becoming compromised. A more proactive focus that links the overall concept of well-being to everyday, concrete actions and activities young people engage in, and that encourages the creation of routine good habits, may lead to better outcomes from online well-being interventions.

## Introduction

Well-being has been shown to be associated with more adaptive responses to negative life events and protection against development of mental health and behavioral problems [1]. Consequently, promotion of well-being has been recognized as a health imperative in many countries worldwide [2-6]. Adolescence and young adulthood (ie, 12-24 years of age) are characterized by significant biological, cognitive, psychological, and social development [7] and influenced by socioeconomic and ecological factors [8]. Mental disorders are a significant disease burden in this age group [9-11]. The prevalence of mental illness in adolescents 12-17 years of age in Australia is 14.4% [12]. It is also a period during which adoption of risky health behaviors, such as tobacco smoking and unsafe sex, most commonly occur [13].

Online well-being interventions have received some attention in the literature as a viable method for improving, at scale, the well-being of young adults [14-18] due to their relevance, accessibility, cost-efficiency, and promotion of anonymity and confidentiality [19-22]. However, in addition to the concepts of well-being varying by age [23], prior research suggests that young people’s concepts of well-being depend on the individual’s aims and values [24]. This is significant, as even professional youth workers can have distinctly different views of well-being compared with those of young people. For example, Bourke and Geldens [25] found that young people viewed the self and relationships as the most important elements to well-being, while youth workers were more focused on social contexts and emotions. Similar research in the field reveals that, overall, younger people tend to place more emphasis on self-knowledge, competence, and self-acceptance, while older people focus more on positively coping with change [26-28]. A more recent study in the United Kingdom that investigated general well-being perceptions of 13-year-olds found that, overwhelmingly, the concept of well-being was linked to the idea of physical health, with very few participants indicating mental health as also being important [29].

Individual conceptualization of well-being also varies according to societal and cultural contexts [8,23]. For example, Chapman [30] questioned how well-being might compete or align with a range of other educative and social goals and agendas, including the achievement of academic outcomes, equity, citizenship, economic prosperity, and social cohesion.

The contested understanding of the term, its substantial increase in use, and its various social meanings make the term *youth well-being* fit for rethinking [31]. Improvements in theorizing and operationalizing youth well-being are likely to occur through strengthening the understanding of the term’s multiple dimensions, based on the views, perspectives, and contexts of young people’s lives [23]. Such knowledge is likely to lead to the design of more relevant and impactful policies and interventions.

This exploratory qualitative study aimed to understand how young people in Australia conceptualize the term *well-being*.

## Methods

### Recruitment and Sampling

The collection of data for this study was conducted collaboratively by two groups of researchers: one group in Sydney, New South Wales, and the other in Adelaide, South Australia. For convenience, and due to the nature of the sites, one site in metropolitan New South Wales (metropolitan 1, M1) and four sites in rural or regional South Australia (rural 1-rural 4, R1-R4) were chosen (see Table 1). One workshop was conducted at each of these five sites.

Participants for the metropolitan workshop were recruited using a recruitment agency and included a mix of young people who were studying, working, and unemployed.  One 5.5-hour workshop was conducted in November 2013.  Participants were offered a small incentive of Aus $50 for their time. The workshop was facilitated by two staff members from the Sydney group.

An additional four workshops were conducted in rural South Australian schools. In order to obtain a dataset reflective of demographics of the selected rural South Australian schools, maximum variation sampling was applied [32]. To achieve this, both public (R2, R3) and private (R4) schools were approached, as well as a school site for disengaged youth (R1). Site R2 was also approached due to its outer regional location. Schools within the selected region were approached via professional contacts (ie, school counselors and year-level coordinators) and the project was advertised within each school site, either via an assembly presentation or during morning announcements. Students were encouraged by teachers to voluntarily participate in the workshop and, as an incentive, were provided with snacks and a certificate of participation. Workshops at the four school sites were run and facilitated between June and July 2014 by two researchers from the Adelaide group.

### The Workshops

Due to logistical constraints, particularly at rural schools, the workshops drew on a range of different methods deemed suitable for the context and participants. All workshops explored how young people think about and experience well-being. In addition, methods were deliberately designed to be open and encourage participants to explore the question from a range of perspectives, including using metaphors for well-being. The rural workshops (R1-R4) were run in a *World Café* style [33]. This style of workshop was chosen due to the interactive nature of this design, which catered well to the age group and classroom setting of the participants, enabling them to respond via written or oral feedback to the group. The length of the sessions ranged from 20 to 30 minutes for each school site. The workshops conducted at schools were shorter due to time constraints associated with school timetables. The following questions were asked: “Well-being—what does it look like?” and “Well-being—what does it feel like?” Students were guided during the workshop to write down and discuss answers to the above questions in small groups.

The metropolitan workshop (M1) allowed for the creation of a shared definition of well-being, mapping well-being goals, and activities that could help to achieve these goals using the photovoice method [34]. Prior to attendance, participants completed a pretask where they took photographs that signified well-being to them and brought them to the workshop. Activities included a discussion and grouping of these photographs, writing down words associated with well-being and creating a shared definition, creating a well-being journey through mapping well-being goals, and exploring what they would need to reach these well-being goals.

**Table 1:** Workshop locations and participant demographics.

Workshop	Details
Metropolitan general young adults (M1)	12 participants (6 male, 6 female); 17-21 years of age; metropolitan New South Wales
Rural disengaged school (R1)	6 participants (5 male, 1 female); 15-22 years of age; inner regional South Australia
Rural public school (R2)	5 participants (5 male); 15-18 years of age; outer regional South Australia
Rural public school (R3)	5 participants (4 male, 1 female); 15-19 years of age; inner regional South Australia
Rural private school (R4)	9 participants (2 male, 7 female); 15-18 years of age; inner regional South Australia

### Data Analysis

All five workshops were audiotaped and the recordings were professionally transcribed. Data from each workshop were collected and analyzed as a whole, with no distinction made between comments or terms expressed by male or female participants or participants of differing ages. Inductive, data-driven coding was then used to analyze the transcript and artifact (ie, written or photographed) data [35]. This *ground-up* approach was chosen so that all data would be coded specifically to identify key themes. The analytic process described by Braun and Clarke [35] was followed; this involved (1) reading and rereading of transcripts, (2) generation of initial codes by manually identifying keywords and phrases in the transcripts and artifacts, (3) searching for themes by grouping similar keywords and phrases and inputting these into an Excel spreadsheet, (4) reviewing themes, (5) defining and naming themes, and (6) producing a report. Steps 1-3 of this process were conducted separately by the two research groups—two authors per group—with the result that each transcript was double coded. Any disagreement between coders was resolved by consensus. The research groups then met in person to review and discuss all data (Step 4) in order to summarize and reach consensus on defining the broader, overarching themes and concepts (Step 5). If the theme emerged in at least four out of five of the workshops, it was classed as a *key theme* and was reported in the Results section.

### Theoretical Framework

Despite using a data-driven, ground-up approach, exploration and analysis of the data were conducted in the context of Keyes’ model of well-being. The researchers coded the key themes based on Keyes’ broad categories of social, emotional, and psychological well-being [36].

## Results

### Themes Underpinning Young People’s Conceptualization of Well-Being

#### Overview

Well-being was found to be a diverse concept and was conceptualized in many different ways; however, similar themes emerged between groups. The seven key themes that emerged were as follows: positive emotions and enjoyable activities, physical wellness, relationships and social connectedness, autonomy and control, goals and purpose, being engaged and challenged, and self-esteem and confidence. These themes are outlined below.

#### Positive Emotions and Enjoyable Activities

In conceptualizing well-being, participants in both groups repeatedly described feelings of happiness and enjoyment. When discussing *happiness*, they described activities related to positive emotions, including *smiles, laughing, seeing the humor in things, making jokes, having a good state of mind,* and *positive attitude*. They also described enjoyable activities that contributed to positive emotions, including *having fun, music, celebrating, doing the things you want to do, shopping, reading, parties, drinking, surfing,* and *gaming*. One participant from the R3 site commented, “If they have hobbies it means they know themselves.”

#### Physical Wellness

In all five workshops, physical wellness was seen as an important aspect of well-being. Participants described the absence of illness (eg, “not going to the hospital”), as well as eating healthily and engaging in physical activity, as important aspects of well-being. Some of the words and activities they described included *health, fitness, healthy eating, fruits and vegetables, exercising, organics, vitamins, swimming, sport, running, sleeping, drinking water, massages,* and *destressing*. One participant from the M1 site commented, “What you do on the outside—your exercise, food—impacts on your mental health.”

#### Social Connectedness and Altruism

Relationships and connections to others played a large role in young people’s understanding of well-being. This occurred at an intrapersonal level (eg, friends and family), as well as at a group (ie, community) level (eg, a football team or club). Participants spoke about *realizing who actually matters, making new friends, unconditional love, loyalty, building relationships,* and *being part of your community*. Such friendships occurred both online and offline and participants did not distinguish between the two. One participant from an R2 site rural school spoke about how it can be easier to make friends and be confident online: “I’m a social butterfly online.”

Participants also spoke about altruism and described how behaviors including *volunteering, respecting others, treating others well, responsibility for your friends, respecting others, kindness, looking after others, thinking of others,* and *caring* can contribute to well-being.

#### Autonomy and Control

Across all groups, participants discussed being independent and in control of their lives and their emotions. They said well-being includes *making decisions, protecting yourself (eg, taekwondo), being in control of yourself, being in control of your life, being in control of your own happiness and your actions, making good choices,* and *rising above.* The following quotes illustrate these sentiments:

Not necessarily in control of their surroundings, but in control of themselves.

R2 site participant

I’d feel independent, like, I’m in control of my own happiness.

R3 site participant

You can’t exactly always rely on other people to make you happy, you have to learn to make yourself happy.

M1 site participant

As part of having autonomy and control, participants discussed having their own money and working. Common themes were *balance between taking time for yourself and study and work, make good choices, “normal” behavior,* and *cleaning*. Money contributed to independence, which was important for well-being, as illustrated in the following quotes:

Saving [money] feels like you’re moving forward.

R4 site participant

Money also goes into part of learning...we're learning to get money, like how to get money, get jobs, we're learning how to respect our money and not just use it all, I guess.

M1 site participant

#### Goals and Purpose

Participants discussed setting and achieving goals and feeling as though they were working toward something. Concepts included *perseverance, being motivated, having hobbies, setting goals, working toward something, planning,* and *hope for the future*.

Achieving and celebrating achievement was important, including *winning a grand final, something to show for your time and effort, recognizing your achievements,* and *receiving awards and prizes*.

Being resilient was also important, particularly in the metropolitan group, as illustrated in the following quote:

Well, if you're feeling down, to be able to get yourself up, get yourself motivated, comes a lot from rugby, really. If you get tackled you have to get yourself up.

M1 site participant

#### Being Engaged and Challenged

A key theme related to well-being was learning new things, trying new things, and challenging yourself in order to grow. Related behaviors included *learning, going to school, being willing to try stuff, keeping your mind occupied, travelling, exploring, seeing new things, discovery, living in the moment, taking risks, learning from your past mistakes, being outside your comfort zone,* and *competition*, as illustrated in the following quote:

...not just comfortable, but challenged by your surroundings, like you’re improving yourself because of them.

R2 site participant

Taking risks was also important to well-being. One participant used the example of going outside your comfort zone to make new friends, as illustrated in the following quote:

Getting friends, you have to take a risk, to go up to them and say, “Hi, my name is blah, blah, blah, blah, blah...” To then hang out with them and all that takes risks, your whole life is about risks.

M1 site participant

#### Self-Esteem and Confidence

Participants felt that a person who possesses well-being has high self-esteem and confidence. Rural groups commented that someone with well-being possesses confident body language and *smiles*. Other behaviors in this theme included *believing in yourself, self-acceptance, being confident, being yourself, focusing on the positives of yourself, free of embarrassment, no judgment,* and *acting on your feelings*.

### Examples of Actions and Things Associated With Maintaining Well-Being—What Makes Good Well-Being Possible?

Participants had no difficulty describing actions and things that lead to good well-being and what they perceived well-being to *feel* and *look* like. Actions that they used to achieve good well-being included physical, emotional, and social activities and are described in Table 2. These actions encompassed *connecting with friends and family, food they enjoyed, focusing on the positives, having employment,* and *leading a fulfilling life*.

### Thinking About Well-Being was Reactive to Stress

Although the young people in the study were able to articulate a complex understanding of well-being when asked, they did not think about their well-being on a day-to-day basis, nor did they generally work to improve it. They did not think about well-being unless there was an issue that impacted negatively on them. Young people thought more about well-being when they were stressed. The following responses were given when a workshop moderator asked the trigger question, “Do you think about your well-being?”

Probably when something really bad happens, is probably when you are more likely to think about yourself.

R4 site participant

...and in challenging situations.

R4 site participant

I feel like in high pressure, as well in Year 12, when things are really full on, you think “Am I sleeping enough? Am I eating enough fruit? Like that kind of thing”...cause you don’t want to just fall over in a heap.

R4 site participant

The following responses were given when a workshop moderator asked the trigger question, “What would prompt you to think about your well-being?”

Whenever I am down, I suppose.

R2 site participant

Let’s face it, crap feelings are always stronger than nice feelings because, let’s face it...you usually do remember crap days.

R2 site participant

**Table 2:** Things perceived to be needed for well-being, identified by young people in the workshops, and organized according to the key themes.

Theme	Examples of actions indicating or leading to well-being	Examples of things needed for well-being
Positive emotions and enjoyable activities	Feeling happy, smiling, laughing, making jokes, positive attitude, having fun, celebrating, shopping, reading, surfing, and gaming	Music, parties, karate, motorbikes, and a good state of mind
Physical wellness	Healthy eating, exercising, swimming, running, sleeping, drinking water, destressing, and relaxing	Health, fitness, sport, massages, organics, vitamins, and fruits and vegetables
Social connectedness and altruism	Talking, accepting others, getting together, loyalty, becoming part of teams or clubs, making new friends, being part of your community, fitting in, volunteering, respecting others, treating others well, responsibility for your friends, kindness, and caring	A support network, friends and family, and unconditional love
Autonomy and control	Protecting yourself, being in control of yourself, being in control of your life, making good choices, rising above, being independent, clearing your mind, letting things go, work-life balance, and “normal” behavior	Safety, stable home life, long drives, freedom, and money
Goals and purpose	Perseverance, being motivated, setting goals, working toward something, planning, hoping for the future, and recognizing your achievements	Receiving awards and prizes, having purpose or a purposeful lifestyle, and having hobbies
Being engaged and challenged	Learning, going to school, travelling, exploring, discovery, living in the moment, taking risks, learning from your past mistakes, being outside comfort zone, and competition	A career
Self-esteem and confidence	Body language, believing in yourself, self-acceptance, being confident, being yourself, being free of embarrassment, and no judgment	Not applicable

The following response was given when a workshop moderator asked the trigger question, “What does it feel like when you don’t have well-being?”

I picture it [well-being] like a plank of wood...when life sucks, it’s splintered, but when it’s not, it’s like smooth, yeah collected.

R4 site participant

### Subthemes

Subthemes that emerged in the rural groups, but did not feature in the metropolitan group, related mainly to the role of place in young people’s lives and the concept of *fitting in*. Rural participants spoke about challenges specific to farming and how they found it difficult when people “from the city judged” their way of life. They commented that although diversity was good, there was also the small-town mentality that being “different is evil.” Themes related to nature (eg, keywords like *the sea, walking on the beach, outdoors, camping, lakes,* and *environment*) occurred only in the metropolitan group and not at all in the rural groups.

## Discussion

### Principal Findings

Although the workshops were conducted in five separate locations, similar themes around how well-being is conceptualized by young people emerged in each group. The findings from this study confirm that well-being is indeed multidimensional, with each of the seven themes identified well-supported by previous research [24,25,37]. Both the pursuit of activities leading to positive experiences that satisfy their desires (ie, hedonic) and a focus on autonomy, purpose, social connectedness, and achieving goals (ie, eudaimonic) contributed to young people’s conceptualizations of well-being [38].

### Comparison With Prior Work

Keyes’ general well-being categories, for example, social, emotional, and psychological well-being [36], were broadly reflected in this study’s results. Our study revealed that young people place high importance on the theme of social connectedness and altruism, which forms part of Keyes’ social well-being construct [36]. This theme is also reflected in Bourke and Geldens’ relationships dimension [25], Armezzani and Paduanello’s relational style of well-being [24], and the relationships component of the *positive emotion, engagement, relationships, meaning,* and *accomplishment* (PERMA) model [37], which emphasizes a person’s relationships with family, friends, colleagues, and community. The workshop participants, especially those from the rural workshops, spoke of connecting with others and maintaining friendships online (eg, via social media or online gaming). This is consistent with prior research on online interactions, which found that the social interactions in online gaming form a considerable element in the enjoyment of playing, with a high percentage of gamers making long-term friends and meeting partners [39].

The importance of physical wellness for well-being among the participants is consistent with Bourke and Geldens’ [25] physical dimensions. This is also consistent with Armezzani and Paduanello’s [24] healthy style of well-being, which focuses on reaching a state of physical balance and avoiding situations that could be a source of physical disorder (eg, smoking, an unhealthy diet, drugs, alcohol, and stress). The workshop participants described physical health as the absence of illness (eg, “not going to the hospital”). These findings highlight how youth-centered understandings of well-being contrast with a disease model, in line with previous research by Graham [40], who found that the discourse around well-being was inherently medical. As a result, what it means to be *well* comes to be defined by the absence of physical symptoms; in other words, to be well is to be not *unwell*. Although themes related to physical health have emerged strongly elsewhere [24], the theme of physical wellness did not particularly dominate in this study. The importance of language in studies of this nature should not be overlooked. In Singletary’s [29] study on young people’s perceptions of mental and physical health, the term *well-being* was not used when surveying participants. Singletary’s findings differ from this study in that only 8% of the young people interviewed perceived being healthy to mean being physically healthy. Another study by Easthope and White [41] found that young people associated the term *health* with things like maintaining a good diet, exercising, and avoiding bad habits, such as smoking and binge drinking; in contrast, *well-being* was strongly associated with social relationships. This suggests that for young people, the terms *well-being* and *health* are conceptualized quite differently, with *well-being* encompassing the broader, holistic human experience and *health* being more limited to physical factors.

This study found that young people generally only think about their well-being in times of stress. Similarly, Bourke and Geldens’ [25] holistic dimension of well-being views well-being as linked to emotional responses to problems that occur in a young person’s life. Similarly, Heady and Wearing [42] propose that stable well-being occurs when individuals have the psychological, social, and physical resources they need to meet a particular challenge. When individuals face more challenges than resources, their well-being is adversely affected [43]. The challenge for programs designed to improve well-being, therefore, is how to help young people address and monitor their well-being before it becomes compromised. This might be achieved by proactively encouraging the development of good habits and increasing resilience by helping young people to think more about caring for their physical and mental health as a matter of routine.

Despite only thinking about their well-being during times of stress, young people in this study were able to give examples of specific actions and things that lead to positive well-being. Honey et al [44] found that young people identified *activities* (eg, study, sport, and parties) and *having things* (eg, food and money) as two important foundations of well-being. Lal et al [45] also found that an important part of the youth experience of well-being was engaging in certain types of activities or *action-oriented states* (eg, exercising). The list of examples described during this study was extensive, stretching across all of the key themes and elements that encompass characterization of well-being.

Linking such key elements of well-being with existing actions and behaviors that young people are familiar with has implications for the design of interventions to promote the active pursuit of well-being. The key to intervention design may be to not promote well-being per se—because clearly young people already know it exists and know what to do to maintain it—but rather to use a strengths-based approach to build on existing practices (eg, running, listening to music, and connecting with friends) in ways that enhance and increase young people’s capacity for well-being. In line with McLeod and Wright [31], findings from our study show that the term *well-being* is a conceptually diverse term for young people. In light of this, it may be more effective for interventions to focus less on the term *well-being* and more on the concrete categories into which elements of well-being can be divided and, in particular, target the actions that can lead to specific improvements. The aims of well-being interventions should perhaps be reframed to resonate with young people’s own views of what they consider as valuable and meaningful ways to achieve and maintain well-being.The finding that themes related to nature (eg, keywords such as *the sea, walking on the beach, outdoors, camping, lakes,* and *environment*) occurred only in the metropolitan group and not at all in the rural groups may simply be because young people in rural areas take being close to nature (eg, more space, more greenery, and smaller population) as a normal part of life; therefore, they may not have felt the need to mention it as something which contributes to their well-being. The metropolitan group may have felt a greater sense or *need* to frequently visit more *natural* places, due to residing in more built-up, less *green* areas. These differences between the rural and metropolitan groups warrant further comparative research. It should also be recognized that the young people from the metropolitan workshop were older on average than those from the rural sites. This could possibly account for the few differences in well-being conceptualization, as the young people were at different stages in their lives (ie, high school and starting university) [29].

### Limitations

This study had a number of limitations. There was an uneven representation of participants from metropolitan and rural sites. As well, the rural participants were primarily sampled from inner regional sites and were, consequently, unrepresentative of young people living in outer regional and remote Australian locations. Therefore, any findings related to location should be understood in this context. In addition, the method of data collection differed between the metropolitan and rural sites with different questions and stimuli used (eg, the metropolitan group was asked to bring in photos while the rural group was not). However, the different methodologies were not compared in terms of their effectiveness, but should rather be viewed as complementary. These differences in approach between the rural and the metropolitan groups reflected logistic difficulties in setting up workshops at the rural schools. In the rural schools, time constraints meant we were only able to have one session with the participants and it was not possible to have them bring photographs to the classroom using the photovoice approach. For this reason, the World Café style of workshop was adopted.

The data were analyzed as a whole for each group, with no distinction made between comments or terms expressed by participants of differing gender or ages; as well, the socioeconomic status of individual participants was not gathered. While it may have been interesting to investigate differences in conceptualization of well-being between male and female participants and between participants of different ages, this was not the primary focus of the study. Despite the slight variations in method and relatively small sample size, the data derived from the workshops were comparable in that they both involved activities designed to generate participants’ concepts of well-being.

### Conclusions

The findings from this study suggest that well-being is a multidimensional concept when conceptualized by young people, with each of the seven themes identified—positive emotions and enjoyable activities, physical wellness, relationships and social connectedness, autonomy and control, goals and purpose, being engaged and challenged, and self-esteem and confidence—being well-supported by previous findings [24,25,36,37]. Young people’s concepts of well-being were diverse and personalized, shaped by ongoing individual contextual experiences related to physical and mental health, along with ecological and social factors.

Since it appears young people think mostly about their well-being in times of stress, the challenge with online well-being interventions is how to get young people to monitor their well-being before it becomes compromised. A more proactive focus may be the key here, that is, linking the overall concept of well-being to everyday, concrete actions or activities young people engage in and encouraging the creation of routine good habits.

The aims and design of online well-being interventions should resonate with young people’s own views. Well-being should be reframed not in terms of a deficit-based response to a problem, but rather as something that can be proactively fostered. Further research could investigate more about what young people would value most in an online well-being intervention and what factors might best trigger its use.

## Abbreviations

PERMA: positive emotion, engagement, relationships, meaning, and accomplishment
